# Association of Unhealthy Lifestyle and Childhood Adversity With Acceleration of Aging Among UK Biobank Participants

**DOI:** 10.1001/jamanetworkopen.2022.30690

**Published:** 2022-09-06

**Authors:** Gan Yang, Xingqi Cao, Xueqin Li, Jingyun Zhang, Chao Ma, Ning Zhang, Qingyun Lu, Eileen M. Crimmins, Thomas M. Gill, Xi Chen, Zuyun Liu

**Affiliations:** 1 School of Public Health and Second Affiliated Hospital, The Key Laboratory of Intelligent Preventive Medicine of Zhejiang Province, Zhejiang University School of Medicine, Hangzhou, Zhejiang, China; 2 School of Economics and Management, Southeast University, Nanjing, Jiangsu, China; 3 Department of Social Medicine School of Public Health and Center for Clinical Big Data and Analytics Second Affiliated Hospital, Zhejiang University School of Medicine, Hangzhou, Zhejiang, China; 4 School of Public Health, Nantong University, Nantong, Jiangsu, China; 5 Davis School of Gerontology, University of Southern California, Los Angeles; 6 Department of Internal Medicine, Yale School of Medicine, New Haven, Connecticut; 7 Department of Health Policy and Management, Yale School of Public Health, New Haven, Connecticut; 8 Department of Economics, Yale University, New Haven, Connecticut

## Abstract

**Importance:**

Accelerated aging makes adults more vulnerable to chronic diseases and death. Whether childhood adversity is associated with accelerated aging processes, and to what extent lifestyle mediates the association, remain unknown.

**Objective:**

To examine the associations of childhood adversity with a phenotypic aging measure and the role of unhealthy lifestyle in mediating these associations.

**Design, Setting, and Participants:**

A retrospective cohort analysis was conducted using data from adult participants in the UK Biobank baseline survey (2006-2010) and online mental health survey (2016). Data analysis was performed from September 1, 2021, to February 28, 2022.

**Exposures:**

Childhood adversity, including physical neglect, emotional neglect, sexual abuse, physical abuse, and emotional abuse, was assessed retrospectively through the online mental health survey (2016).

**Main Outcomes and Measures:**

A phenotypic aging measure, phenotypic age acceleration, was calculated, with higher values indicating accelerated aging. Body mass index, smoking status, alcohol consumption, physical activity, and diet were combined to construct an unhealthy lifestyle score (range, 0-5, with higher scores denoting a more unhealthy lifestyle).

**Results:**

A total of 127 495 participants aged 40 to 69 years (mean [SD] chronological age at baseline, 56.4 [7.7] years; 70 979 women [55.7%]; 123 987 White participants [97.2%]) were included. Each individual type of childhood adversity and cumulative childhood adversity score were associated with phenotypic age acceleration. For instance, compared with participants who did not experience childhood adversity, those who experienced 4 (β = 0.296, 95% CI, 0.130-0.462) or 5 (β = 0.833; 95% CI, 0.537-1.129) childhood adversities had higher phenotypic age acceleration in fully adjusted models. The formal mediation analysis revealed that unhealthy lifestyle partially mediated the associations of childhood adversity with phenotypic age acceleration by 11.8% to 42.1%.

**Conclusions and Relevance:**

In this retrospective cohort study, childhood adversity was significantly associated with acceleration of aging and, more importantly, unhealthy lifestyle partially mediated these associations. These findings reveal a pathway from childhood adversity to health in middle and early older adulthood through lifestyle and underscore the potential of more psychological strategies beyond lifestyle interventions to promote healthy aging.

## Key Points

**Question:**

Is childhood adversity associated with acceleration of aging and, if so, does lifestyle mediate the association?

**Findings:**

In this cohort study of 127 495 adults aged 40 to 69 years in the UK Biobank, childhood adversity was significantly associated with acceleration of phenotypic aging, and unhealthy lifestyle partially mediated the associations by 11.8% to 42.1%.

**Meaning:**

These findings reveal a pathway from childhood adversity to health in middle and early older adulthood through modifiable lifestyle and underscore the potential of more psychological strategies beyond lifestyle interventions to promote healthy aging.

## Teaser

This cohort study examines the associations of childhood adversity with a phenotypic aging measure and the role of unhealthy lifestyle in mediating these associations.

## Introduction

Aging is a complex process of multisystem physiological dysregulation, and accelerated aging makes adults more vulnerable to chronic diseases and death.^1^ Delaying aging has been a goal of human beings over a long history. Understanding the factors associated with aging may help us to work toward addressing racial and ethnic disparities in aging and creating lifespan equality. Meanwhile, how to estimate the outcomes of interventions to delay aging remains a challenge given the lack of comprehensive aging measures. Chronological aging serves as the major factor associated with risk of many chronic diseases and death. However, persons with the same chronological age may differ in their rates of aging. We have developed a novel phenotypic aging measure—phenotypic age acceleration—using 9 clinical biomarkers chosen for their ability to estimate mortality and morbidity.^2,3^ This aging measure provides a useful indicator for evaluating the outcomes of geroprotective interventions, identification of risk factors, and elucidation of mechanisms of aging.^4^

A number of studies^5,6,7^ have documented the association of early life factors such as childhood adversity with health in late life. Given that chronological aging serves as the major factor associated with risk of late-life chronic diseases and death, it follows that childhood adversity may accelerate the aging process and then place persons at higher risk of chronic diseases. A few studies have explored the associations of childhood adversity with telomere length^8^ and DNA methylation–based accelerated aging.^9,10^ Given that phenotypic age acceleration outperforms aging measures at the molecular level (eg, telomere length) in estimating adverse health outcomes,^11,12,13^ it is necessary to evaluate the association of childhood adversity with phenotypic age acceleration.

Of note, how childhood adversity affects accelerated aging remains unknown. Evidence from recent theoretical and empirical studies suggests that childhood adversity has a long-term association with high-risk behaviors,^14^ including behavior associated with a high risk of HIV infection, depression, and unhealthy lifestyle, thus, affecting health in late life. For instance, Anda et al^15^ have reported that persons who experienced sexual abuse had nearly 3 times the odds of smoking compared with those who did not, resulting in high risks of cancer, pulmonary diseases, and other adverse outcomes. Meanwhile, aging is strongly responsive to modifiable lifestyle factors (eg, smoking, alcohol consumption, and physical activities).^16,17^ Therefore, we hypothesize that unhealthy lifestyles may partially mediate the association of childhood adversity with accelerated aging, which has not been investigated in previous research.

We conducted this study using data from UK Biobank (UKB), a large population-based cohort study with approximately 500 000 participants aged 40 to 69 years.^18^ This study aimed to examine the associations of childhood adversity with phenotypic age acceleration, as well as the role of unhealthy lifestyle as a mediator in the associations.

## Methods

### Study Participants

This was a retrospective cohort study leveraging the UKB data. The baseline survey of UKB was conducted in 2006 to 2010, and 499 309 participants were recruited. In 2016, almost two-thirds of the participants were chosen to conduct an online mental health questionnaire. The 156 749 individuals who participated in both the baseline survey and online mental health survey were eligible for our study.^19^ We excluded participants with missing data on childhood adversity (3728 participants) and clinical biomarkers used to calculate phenotypic age acceleration (25 526 participants) (eFigure in the Supplement). The UKB study was approved by the North West Multi-Centre Research Ethics Committee as a Research Tissue Bank. Written informed consent was provided by each participant before the study, and researchers are allowed to use data from UKB without an additional ethical clearance. This study followed the Strengthening the Reporting of Observational Studies in Epidemiology (STROBE) reporting guidelines.

### Assessment of Childhood Adversity

Childhood adversity, sourced from the 2016 online mental health questionnaire survey, was assessed with 5 questions representing physical neglect, emotional neglect, sexual abuse, physical abuse, and emotional abuse, using the Childhood Trauma Screener: (1) someone to take to doctor when needed as a child (physical neglect); (2) felt loved as a child (emotional neglect); (3) sexually molested as a child (sexual abuse); (4) physically abused by family as a child (physical abuse); or (5) felt hated by a family member (emotional abuse).^20^

The Childhood Trauma Screener, a shortened version of the Childhood Trauma Questionnaire, is a cost-efficient, validated, and relatively reliable screening tool in large epidemiological studies.^21^ All items were referred to as, “When I was growing up (age <16 years old).” For each question, potential responses included never true, rarely true, sometimes true, often true, and very often true. Physical neglect was dichotomized as 1 if participants answered never true, rarely true, sometimes true, or often true; emotional neglect was dichotomized as 1 if participants answered never true, rarely true, or sometimes true; sexual abuse, physical abuse, and emotional abuse were dichotomized as 1 if participants answered rarely true, sometimes true, often true, and very often true^22^. The summary score of 5 items ranged from 0 to 5, with a higher score denoting more childhood adversities. The details of questions and cutoff points are shown in eTable 1 in the Supplement.

### Assessment of Lifestyle

As described in previous studies,^23^ an unhealthy lifestyle score was established by 5 lifestyle factors, including body mass index (calculated as weight in kilograms divided by height in meters squared), smoking status, alcohol consumption, physical activity, and diet. The data on lifestyle factors were collected by structured questionnaires and 24-hour dietary recall.

According to the recommendations from the World Health Organization,^24^ having a body mass index lower than 18.5 or higher than 24.9 was considered as unhealthy. Smoking 100 cigarettes or more in life was classed as ever smoking and thus as unhealthy.^23^ According to the guidelines in the UK, the daily consumption of more than 1 drink for women and more than 2 drinks for men was considered as unhealthy.^23^ For physical activity, engaging in vigorous activity less than 75 minutes per week or once a week was defined as unhealthy, or engaging in moderate physical activity less than 150 minutes per week or 5 days a week was considered as unhealthy.^25^ For diet, unhealthy was defined as having not achieved the intake goals for more than half of the following components: fruits, vegetables, fish and shellfish, dairy products, whole grains, vegetable oils, refined grains, sugar-sweetened beverages, and unprocessed meats.^26^ The details of the intake goals of each diet component have been published elsewhere.^26,27^

For each lifestyle factor, an unhealthy level was scored 1 and, otherwise, was scored 0. The unhealthy lifestyle score used as a continuous variable was defined as the summary score of 5 lifestyle factors, with a range of 0 to 5. A higher score indicated a higher level of unhealthy lifestyle.

### Phenotypic Age Acceleration

The biomarkers used to determine phenotypic age acceleration were obtained from biological samples at the time of participants enrollment.^28^ The samples were typically analyzed at the UK Biobank central laboratory within 24 hours of blood draw with Beckman Coulter LH750 instruments, and the laboratory results were archived in the participants’ data files. Details of biomarker data processing can be found on the website of UK Biobank.^29,30^

Phenotypic age was developed by regressing the hazard of mortality on 42 clinical biomarkers and chronological age.^2,3^ Finally, 9 clinical biomarkers and chronological age at baseline were selected into a parametric proportional hazards model based on the Gompertz distribution and then we converted 10-year mortality risk into units of years. The formula of phenotypic age is presented as follows:



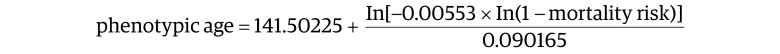



where



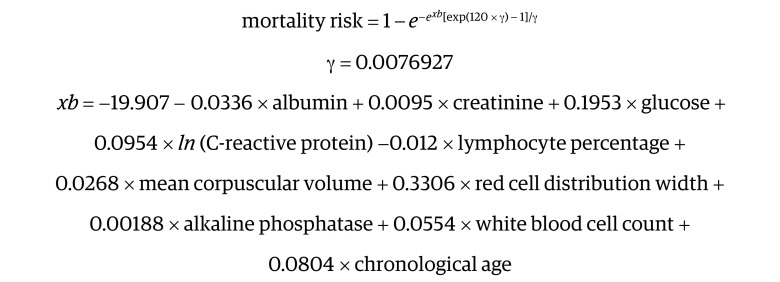



The phenotypic age equation has been widely used in literature over recent years, and it shows that phenotypic age captures morbidity and mortality risk in many populations from different countries.^31,32^ Phenotypic age acceleration was calculated as a residual of phenotypic age adjusted for chronological age by linear regression. Participants with phenotypic age acceleration value greater than 0 were defined as phenotypically older, whereas those with phenotypic age acceleration value less than 0 were defined as phenotypically younger. The detailed description of the phenotypic age acceleration has been published elsewhere.^2,3^

### Covariates

Covariates included chronological age at baseline, sex, race and ethnicity, educational level, occupation, Townsend Deprivation Index (TDI), maternal smoking, and history of cardiovascular disease (CVD) and cancer, which were collected at baseline. Race and ethnicity were included because of their potential confounding or differences and were defined as Black, Chinese, multiple races or ethnicities, South Asian, White, and other (ie, any other race or ethnicity not already specified) as reported by the participants.^33^ Educational level was classified as high (college or university degree), intermediate (advanced [A/AS] levels or ordinary level [O-levels], general certificate of secondary education, or equivalent, equivalent to grades 6-12 in the US school system), and low (none of the aforementioned).^23^ Occupation was classified as working, retired, and other (unpaid or voluntary work, full-time or part-time student, looking after home and/or family, unable to work because of sickness or disability, unemployed, or did not answer).^34^ TDI used census data on employment, housing, and social class based on the postal code of participants.^35^ For the TDI, 0 indicates the mean value for an area, positive numbers indicate lower socioeconomic status, and negative numbers indicate higher socioeconomic status. We also considered history of disease (ie, CVD and cancer) in this study, which was dichotomized as yes or no.

### Statistical Analysis

We described the basic characteristics of the participants with mean (SD) for continuous variables and count (percentage) for categorical variables, respectively. Multiple imputations by chained equations^36^ were used to impute missing values of smoking (1258 participants), alcohol consumption (13 690 participants), diet (1 participant), physical activity (661 participants), and covariates (16 899 participants).

**Figure 1.**
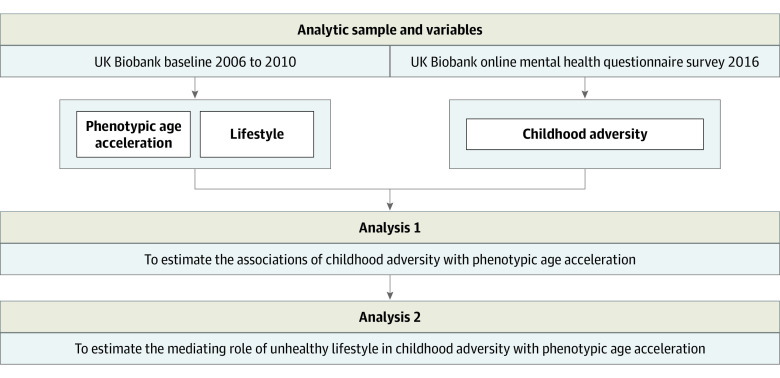
Roadmap for Evaluating Associations Between Childhood Adversity, Unhealthy Lifestyle, and Phenotypic Age Acceleration

The analytic plan for this study is presented in Figure 1. We used phenotypic age acceleration as the primary outcome and childhood adversity as well as unhealthy lifestyle score as the variables. First, general linear regression models were performed to examine the associations of childhood adversity with phenotypic age acceleration. We documented coefficients and corresponding 95% CIs from 3 models. Model 1 was adjusted for sex. Model 2 was additionally adjusted for race and ethnicity, educational level, occupation, TDI, maternal smoking, and history of CVD and cancer. Model 3 was additionally adjusted for unhealthy lifestyle score.

Second, to investigate whether an unhealthy lifestyle mediates the associations of childhood adversity with phenotypic age acceleration, we performed the following analyses in addition to the aforementioned general linear regression models. First, general linear regression models were estimated to examine the associations of childhood adversity with unhealthy lifestyle score in 2 models. Model 1 was adjusted for chronological age and sex. Model 2 further was adjusted for race and ethnicity, educational level, occupation, TDI, maternal smoking, and history of CVD and cancer. Next, the mediation analysis was performed with the R package mediation with 1000 simulations. The mediation proportions and corresponding 95% CIs were documented after adjustment for sex. We repeated the above analysis stratified by chronological age (40-59 vs 60-69 years) and sex. To test the robustness of the findings, we first compared the basic characteristics of participants excluded because of missing data for clinical biomarkers (used to calculate phenotypic age acceleration) and the total population who participated in both the baseline survey and online mental health survey. Then, we repeated the main analysis first using childhood adversity as a continuous variable (range, 0-20) and second using a complete-case sample of 95 273 participants.

All analyses were conducted using SAS statistical software version 9.4 (SAS Institute) and R statistical software version 4.1.1 (R Project for Statistical Computing). Two-tailed *P* < .05 was considered significant. Data analysis was performed from September 1, 2021, to February 28, 2022.

## Results

### Baseline Characteristics of Participants

**Figure 2.**
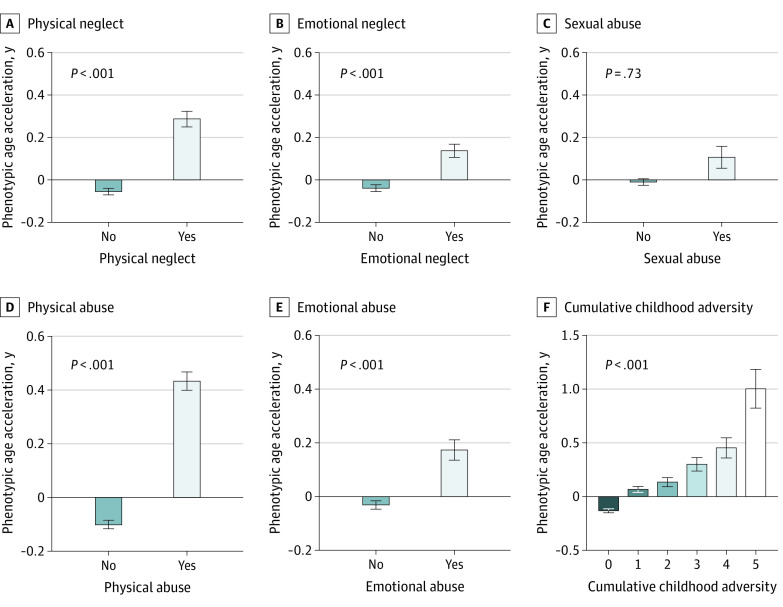
Phenotypic Age Acceleration by Type of Childhood Adversity

The baseline characteristics of the 127 495 participants are presented in Table 1. The mean (SD) chronological age of the participants was 56.4 (7.7) years; 70 979 participants (55.7%) were women, and 123 987 (97.2%) were White. Figure 2 presents the mean (SE) level of phenotypic age acceleration in subgroups of childhood adversity. Compared with participants who did not experience childhood adversity, those who experienced physical neglect, emotional neglect, physical abuse, or emotional abuse had higher phenotypic age acceleration (for physical neglect, mean [SE], −0.06 [0.02] vs 0.29 [0.04] years; *P* < .001).

**Table 1.** Baseline Characteristics of the Study Participants

Characteristics	Participants, No. (%) (N = 127 495)
Chronological age, mean (SD), y	56.4 (7.7)
Phenotypic age, mean (SD), y	52.0 (9.3)
Sex	
Female	70 979 (55.7)
Male	56 516 (44.3)
Race and ethnicity	
Black	853 (0.7)
Chinese	288 (0.2)
South Asian	1020 (0.8)
White	123 987 (97.2)
Multiple	652 (0.5)
Other^a^	695 (0.5)
Educational level^b^	
High	59 057 (46.3)
Intermediate	41 982 (32.9)
Low	26 456 (20.8)
Occupation	
Working	81 358 (63.8)
Retired	37 731 (29.6)
Other	8406 (6.6)
Townsend Deprivation Index, mean (SD)^c^	−1.7 (2.8)
Body mass index, mean (SD)^d^	26.8 (4.5)
≤18.5	721 (0.6)
18.5-24.9	47 324 (37.1)
≥24.9	79 450 (62.3)
Never smoking	73 859 (57.9)
Never drinking	76 732 (60.2)
Regular exercise	94 688 (74.3)
Healthy diet	49 163 (38.6)
Maternal smoking around birth, yes	36 704 (28.8)
Prevalent cardiovascular disease, yes	5590 (4.4)
Prevalent cancer, yes	9811 (7.7)

^a^ Other includes any races or ethnicities not otherwise specified.

^b^ High educational level is defined as having a college or university degree. Intermediate educational level is defined as advanced (A/AS) levels or equivalent, ordinary level (O-level), general certificate of secondary education, or equivalent. Intermediate educational levels refer to grades 6 to 12 (O-level equals middle school or junior high, grades 6-8; A/AS-level equals high school, grades 9-12) in the US school system. Low educational level is defined as none of the aforementioned.

^c^ For the Townsend Deprivation Index, 0 indicates the mean value for an area, positive numbers indicate lower socioeconomic status, and negative numbers indicate higher socioeconomic status.

^d^ Body mass index is calculated as weight in kilograms divided by height in meters squared.

### Associations of Childhood Adversity With Phenotypic Age Acceleration

Table 2 shows the associations of childhood adversity with phenotypic age acceleration. For instance, compared with participants who did not experience any childhood adversity, those who experienced physical neglect had an increase in phenotypic age acceleration (β = 0.415; 95% CI, 0.340-0.490) after adjusting for sex (model 1). After further adjustment for more covariates, including race and ethnicity, educational level, occupation, TDI, maternal smoking around birth, and history of CVD and cancer, these associations were maintained (model 2). The associations were reduced but remained significant after additionally adjusting for the unhealthy lifestyle score (model 3). Also, for cumulative childhood adversity score, we observed dose-response associations (β = 0.076; 95% CI, 0.051-0.100) in model 3. In the fully adjusted model, participants who experienced 4 (β = 0.296; 95% CI, 0.130-0.462) or 5 (β = 0.833; 95% CI, 0.537-1.129) childhood adversities had higher phenotypic age acceleration, compared with those who did not experience adversity.

**Table 2.** Associations of Childhood Adversity With Phenotypic Age Acceleration and Mediation Proportion of Childhood Adversity in Phenotypic Age Acceleration Attributed to Unhealthy Lifestyle

Childhood adversity	β (95% CI)			Mediation proportion, % (95% CI)^d^	*P* value
	Model 1^a^	Model 2^b^	Model 3^c^		
Physical neglect	0.415 (0.340 to 0.490)	0.233 (0.157 to 0.308)	0.221 (0.146 to 0.296)	11.8 (8.8 to 16.0)	<.001
Emotional neglect	0.206 (0.139 to 0.273)	0.086 (0.020 to 0.153)	0.012 (−0.054 to 0.077)	52.1 (39.3 to 77.0)	<.001
Sexual abuse	0.377 (0.278 to 0.475)	0.278 (0.180 to 0.376)	0.174 (0.077 to 0.271)	34.8 (27.5 to 47.0)	<.001
Physical abuse	0.418 (0.347 to 0.489)	0.302 (0.231 to 0.373)	0.199 (0.128 to 0.269)	32.2 (27.1 to 39.0)	<.001
Emotional abuse	0.349 (0.272 to 0.426)	0.222 (0.145 to 0.299)	0.124 (0.048 to 0.201)	36.9 (30.3 to 48.0)	<.001
Cumulative childhood adversity score (0-5)	0.186 (0.161 to 0.211)	0.117 (0.092 to 0.142)	0.076 (0.051 to 0.100)	NA	NA
0	1 [Reference]	1 [Reference]	1 [Reference]	NA	NA
1	0.164 (0.098 to 0.231)	0.100 (0.034 to 0.166)	0.065 (−0.001 to 0.130)	31.4 (21.1 to 53.0)	<.001
2	0.272 (0.182 to 0.362)	0.147 (0.057 to 0.237)	0.064 (−0.025 to 0.153)	42.1 (31.5 to 65.0)	<.001
3	0.523 (0.402 to 0.645)	0.331 (0.209 to 0.452)	0.200 (0.080 to 0.321)	34.1 (26.9 to 45.0)	<.001
4	0.726 (0.558 to 0.894)	0.457 (0.289 to 0.624)	0.296 (0.130 to 0.462)	30.7 (24.1 to 40.0)	<.001
5	1.488 (1.189 to 1.788)	1.050 (0.751 to 1.349)	0.833 (0.537 to 1.129)	20.9 (16.5 to 27.0)	<.001

Abbreviation: NA, not applicable.

^a^ Model 1 was adjusted for sex.

^b^ Model 2 was further adjusted for race and ethnicity, educational level, occupation, Townsend Deprivation Index, maternal smoking around birth, and history of cardiovascular disease and cancer based on model 1.

^c^ Model 3 was further adjusted for unhealthy lifestyle score based on model 2.

^d^ The model included sex and unhealthy lifestyle score.

### Mediation Analyses of Unhealthy Lifestyle in the Association of Childhood Adversity With Phenotypic Age Acceleration

First, the association between childhood adversity and unhealthy lifestyle is shown in Table 3. In the fully adjusted model (ie, model 2), each individual type of childhood adversity and cumulative childhood adversity score were positively associated with unhealthy lifestyle score. For instance, compared with participants who did not experience any childhood adversity, those who experienced 4 (β = 0.260; 95% CI, 0.221-0.298) or 5 (β = 0.350; 95% CI, 0.281-0.419) childhood adversities had an increased unhealthy lifestyle score.

**Table 3.** Associations of Childhood Adversity With Unhealthy Lifestyle Score

Childhood adversity	β (95% CI)	
	Model 1^a^	Model 2^b^
Physical neglect	0.072 (0.055-0.090)	0.019 (0.001-0.036)
Emotional neglect	0.158 (0.142-0.173)	0.120 (0.104-0.135)
Sexual abuse	0.196 (0.173-0.219)	0.167 (0.144-0.190)
Physical abuse	0.199 (0.183-0.216)	0.166 (0.150-0.183)
Emotional abuse	0.191 (0.174-0.209)	0.157 (0.139-0.175)
Cumulative childhood adversity score (0-5)	0.087 (0.081-0.093)	0.067 (0.062-0.073)
0	1 [Reference]	1 [Reference]
1	0.077 (0.062-0.093)	0.057 (0.042-0.073)
2	0.173 (0.152-0.193)	0.134 (0.113-0.154)
3	0.266 (0.238-0.294)	0.210 (0.182-0.238)
4	0.334 (0.295-0.372)	0.260 (0.221-0.298)
5	0.466 (0.397-0.536)	0.350 (0.281-0.419)

^a^ Model 1 was adjusted for chronological age and sex.

^b^ Model 2 was further adjusted for race and ethnicity, educational level, occupation, Townsend Deprivation Index, and maternal smoking around birth, and history of cardiovascular disease and cancer based on model 1.

Second, the association of unhealthy lifestyle with phenotypic age acceleration is presented in eTable 2 in the Supplement. With a 1-point increase in unhealthy lifestyle score, phenotypic age acceleration increased by a β value of 0.68 (95% CI, 0.66-0.70) (model 1). After adjusting for other covariates, the results remained unchanged in model 2.

Finally, as shown in Table 2, an unhealthy lifestyle partially mediated the associations of childhood adversity with phenotypic age acceleration. Compared with participants experiencing no childhood adversity, unhealthy lifestyle partially mediated 11.8% to 42.1% of phenotypic age acceleration for those who experienced some childhood adversity.

### Additional Analyses

First, eTable 3 in the Supplement shows the analyses stratified by chronological age. The associations between cumulative childhood adversity score and phenotypic age acceleration were significant, and there were no interactions of cumulative childhood adversity score with chronological age. Second, the stratification analyses showed interactions of some individual types of childhood adversity and cumulative childhood adversity score with sex on phenotypic age acceleration (eTable 4 in the Supplement). For example, the associations of cumulative childhood adversity score with phenotypic age acceleration were more pronounced in men (β = 0.081; 95% CI, 0.041-0.120) than in women (β = 0.066; 95% CI, 0.034-0.098; *P* for interaction < .001). Third, we found that participants excluded because of missing data for clinical biomarkers were more likely to be women and less likely to be White, compared with the total population who participated the baseline survey and online mental health survey (eTable 5 in the Supplement). Fourth, our sensitivity analysis showed similar results when using childhood adversity as a continuous variable, or using a complete-case sample, respectively (eTable 6 and eTable 7 in the Supplement).

## Discussion

In this cohort study of an almost entirely White population of 127 495 adults aged 40 to 69 years in the UKB, we found that childhood adversity was associated with acceleration of phenotypic aging. More importantly, we demonstrated that unhealthy lifestyle partially mediated the associations. The findings highlight the importance of reducing traumatic experiences in early life. Furthermore, the findings reveal a pathway linking childhood adversity to aging and suggest the potential of lifestyle interventions as well as other strategies to slow aging among adults who have already experienced childhood adversity.

Few studies have explored the association between childhood adversity and phenotypic age acceleration. Our previous study^37^ conducted in the US population found that childhood adversity contributed to partial variance in phenotypic age acceleration, which was consistent with the findings of the current study. Given that each 1-year increase of phenotypic age acceleration increases the risk of mortality by 9%,^3^ the robust findings of the associations between childhood adversity and phenotypic aging have important public implications for interventions on adverse childhood experiences in early life to improve health and diminish health inequality in middle and early older adulthood.

The findings that unhealthy lifestyle partially mediated the associations of childhood adversity with phenotypic age acceleration provide clues for mechanisms linking childhood adversity to aging. In addition to chronic stress caused by childhood adversity,^38^ we suggest that individuals who experienced childhood adversity might be more likely to adopt unhealthy lifestyles that are socially patterned (eg, poor diet, smoking, or drinking) as a way to avoid or reduce stress, which may potentially lead to phenotypic aging in the long term.^39^ In fact, childhood adversity has been associated with a high risk of alcoholism, smoking, physical inactivity, and severe obesity.^40^ Several studies have demonstrated that adherence to a healthy lifestyle may slow phenotypic aging.^41,42^ In particular, a secondary analysis of a randomized clinical trial has suggested that caloric restriction has slowed aging.^41^ This confirms our hypothesis that childhood adversity leads to unhealthy lifestyle, which, in turn, leads to accelerated phenotypic aging. Our findings extend previous studies^43^ on individual lifestyle factors, such as smoking or other substance use behaviors, which have been shown to mediate the association between childhood poverty–related stress and allostatic load. Furthermore, the findings of mediation analysis highlight the importance of lifestyle interventions to mitigate the accelerated aging process. More importantly, the partial mediation suggests that among adults who have already experienced childhood adversity, programs that provide lifestyle interventions should address the psychological effects of childhood adversity, as well as teaching healthy lifestyle skills. However, given that individuals who were more neurotic (which is well known be associated with adversity and unhealthy lifestyle) than average may recall more adversities,^44^ further research is needed to examine the association between childhood adversity, unhealthy lifestyle, aging, and psychological and psychiatric factors. The findings of the mediation analyses should be interpreted cautiously because the statistical mediation observed did not necessarily represent a prospective mediation.

The consistent results of childhood adversity with phenotypic age acceleration in population subgroups strengthen our findings. Of note, we found an interaction between cumulative childhood adversity score and phenotypic age acceleration stratified by sex. Cumulative adversity score was more associated with premature aging among men than women. This finding is complex given the sex differences in hormones, psychology, and developmental speed,^45^ and further studies focused on sex differences are needed in the future.

### Strengths and Limitations

The present study has some strengths, including the large population of middle-aged and older adults and a series of additional analyses that were performed to confirm the validity of the findings. This study also has limitations. First, older adults may not accurately recall childhood experiences, resulting in age-dependent memory bias. Also, self-reports of outcomes in late life may be colored by some adults who have an extremely negative view of their childhood experiences.^44^ Thus, further prospective cohort studies and objective measurements are urgently needed in this field. Second, our study did not assess the severity and the duration of childhood adversity. In moving forward, further studies should consider multiple aspects of adversity, including the age when adversity first occurred, to reinforce the findings of our study. Third, potential survivor bias may exist. Fourth, participants in our study were mostly White and were healthier and had higher socioeconomic status than the general population in UK. Therefore, the findings may not be generalizable to the general population.

## Conclusions

In summary, among this almost entirely White population of 127 495 adults aged 40 to 69 years old in UKB, childhood adversity was significantly associated with acceleration of aging. Furthermore, unhealthy lifestyle partially mediated the associations. The findings call for more attention to childhood adversity in young children and teenagers. More importantly, the findings reveal a pathway linking childhood adversity to health in middle and early older adulthood through aging and underscore the potential of lifestyle intervention as well as other strategies to promote healthy aging among adults who have already experienced childhood adversity.

## Notes

Supplement. **eFigure.** Flow Chart of the Analytic Sample **eTable 1.** Questions and Responses for Variables Included in the Childhood Adversity in UKB **eTable 2.** Associations of Unhealthy Lifestyle With Phenotypic Age Acceleration **eTable 3.** Associations Between Childhood Adversity and Phenotypic Age Acceleration by Chronological Age **eTable 4.** Associations Between Childhood Adversity and Phenotypic Age Acceleration by Sex **eTable 5.** Baseline Characteristics of Participants Excluded Because of Missing Data on Clinical Biomarkers (Used to Calculate Phenotypic Age Acceleration) and the Total Population Who Participated in Both the Baseline Survey and Online Mental Health Survey **eTable 6.** Associations of Childhood Adversity (as a Continuous Variable, Range 0-20) With Phenotypic Age Acceleration and Mediation Proportion of Childhood Adversity in Phenotypic Age Acceleration Attributed to Unhealthy Lifestyle ** eTable 7.** Associations of Childhood Adversity With Phenotypic Age Acceleration and Mediation Proportion of Childhood Adversity in Phenotypic Age Acceleration Attributed to Unhealthy Lifestyle in a Complete-Case Sample (N=95,273)
