# Supplementary material for: Association of Unhealthy Lifestyle and Childhood Adversity With Acceleration of Aging Among UK Biobank Participants
Source: JAMA Netw Open. 2022 Sep 6;5(9):e2230690. doi: 10.1001/jamanetworkopen.2022.30690 (PMC9449787; doi:10.1001/jamanetworkopen.2022.30690)
Supplement: Supplement. — eFigure. Flow Chart of the Analytic Sample eTable 1. Questions and Responses for Variables Included in the Childhood Adversity in UKB eTable 2. Associations of Unhealthy Lifestyle With Phenotypic Age Acceleration eTable 3. Associations Between Childhood Adversity and Phenotypic Age Acceleration by Chronological Age eTable 4. Associations Between Childhood Adversity and Phenotypic Age Acceleration by Sex eTable 5. Baseline Characteristics of Participants Excluded Because of Missing Data on Clinical Biomarkers (Used to Calculate Phenotypic Age Acceleration) and the Total Population Who Participated in Both the Baseline Survey and Online Mental Health Survey eTable 6. Associations of Childhood Adversity (as a Continuous Variable, Range 0-20) With Phenotypic Age Acceleration and Mediation Proportion of Childhood Adversity in Phenotypic Age Acceleration Attributed to Unhealthy Lifestyle eTable 7. Associations of Childhood Adversity With Phenotypic Age Acceleration and Mediation Proportion of Childhood Adversity in Phenotypic Age Acceleration Attributed to Unhealthy Lifestyle in a Complete-Case Sample (N=95,273) [file jamanetwopen-e2230690-s001.pdf]

Supplemental Online Content

Yang G, Cao X, Li X, et al. Association of unhealthy lifestyle and childhood adversity with acceleration of aging among UK Biobank participants. *JAMA Netw Open*. 2022;5(9):e2230690.
doi:10.1001/jamanetworkopen.2022.30690

eFigure. Flow Chart of the Analytic Sample
eTable 1. Questions and Responses for Variables Included in the Childhood Adversity in UKB
eTable 2. Associations of Unhealthy Lifestyle With Phenotypic Age Acceleration
eTable 3. Associations Between Childhood Adversity and Phenotypic Age Acceleration by Chronological Age
eTable 4. Associations Between Childhood Adversity and Phenotypic Age Acceleration by Sex
eTable 5. Baseline Characteristics of Participants Excluded Because of Missing Data on Clinical Biomarkers (Used to Calculate Phenotypic Age Acceleration) and the Total Population Who Participated in Both the Baseline Survey and Online Mental Health Survey
eTable 6. Associations of Childhood Adversity (as a Continuous Variable, Range 0-20) With Phenotypic Age Acceleration and Mediation Proportion of Childhood Adversity in Phenotypic Age Acceleration Attributed to Unhealthy Lifestyle
eTable 7. Associations of Childhood Adversity With Phenotypic Age Acceleration and Mediation Proportion of Childhood Adversity in Phenotypic Age Acceleration Attributed to Unhealthy Lifestyle in a Complete-Case Sample (N=95,273)

This supplemental material has been provided by the authors to give readers additional information about their work.

© 2022 Yang G et al. *JAMA Network Open.*

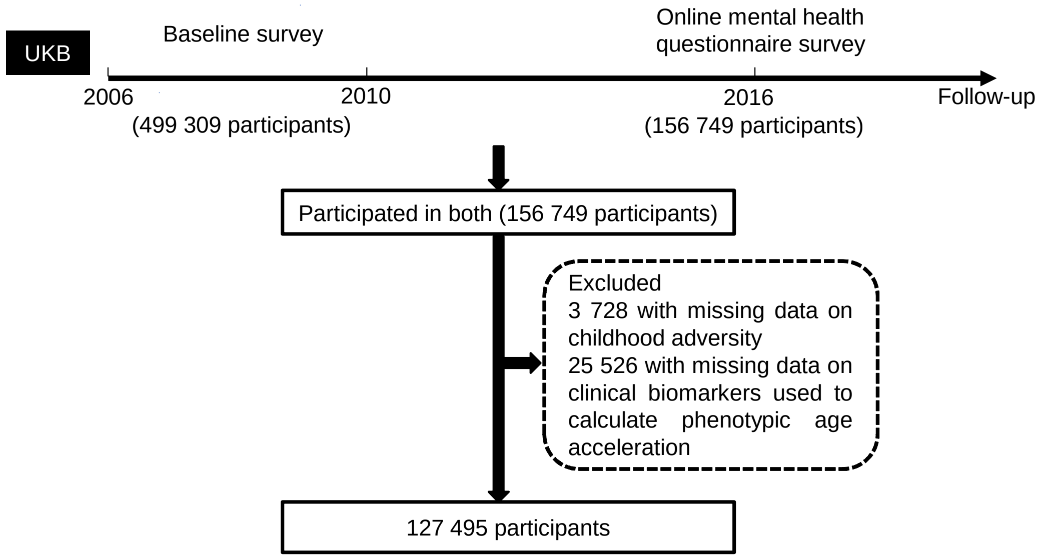

eFigure. Flow Chart of the Analytic Sample

© 2022 Yang G et al. *JAMA Network Open.*

eTable 1. Questions and Responses for Variables Included in the Childhood Adversity in UKB

Childhood adversity questions		Responses/description	Cut off
Physical neglect	Someone to take to doctor when needed as a child.	0, Never true; 1, Rarely true; 2, Sometimes true; 3, Often; 4, Very often true	≤3
Emotional neglect	Felt loved as a child.	0, Never true; 1, Rarely true; 2, Sometimes true; 3, Often; 4, Very often true	≤2
Sexual abuse	Sexually molested as a child.	0, Never true; 1, Rarely true; 2, Sometimes true; 3, Often; 4, Very often true	≥1
Physical abuse	Physically abused by family as a child.	0, Never true; 1, Rarely true; 2, Sometimes true; 3, Often; 4, Very often true	≥1
Emotional abuse	Felt hated by family member as a child.	0, Never true; 1, Rarely true; 2, Sometimes true; 3, Often; 4, Very often true	≥1
Cumulative childhood adversity risk	Summary score of childhood adversity.	Summary score of five childhood adversity items. (0-5)	—

© 2022 Yang G et al. *JAMA Network Open.*

eTable 2. Associations of Unhealthy Lifestyle With Phenotypic Age Acceleration

	**Model 1**	**Model 2**
	**β (95% CI)**	**β (95% CI)**
Unhealthy lifestyle score	0.68 (0.66, 0.70)	0.62 (0.60, 0.65)

Abbreviations: CI, confidence interval.
Notes:
Model 1: adjusted for sex.
Model 2: further adjusted for race and ethnicity, educational level, occupation, Townsend deprivation index, maternal smoking around birth, and history of cardiovascular disease and cancer based on Model 1.

© 2022 Yang G et al. *JAMA Network Open.*

eTable 3. Associations Between Childhood Adversity and Phenotypic Age Acceleration by Chronological Age

Childhood adversity	Model 1	Model 2	Model 3	P for interaction
	β (95% CI)	β (95% CI)	β (95% CI)	
Physical neglect				0.12
40-59 years	0.529 (0.422, 0.635)	0.323 (0.216, 0.430)	0.281 (0.176, 0.387)	
60-69 years	0.266 (0.160, 0.372)	0.159 (0.053, 0.266)	0.163 (0.057, 0.268)	
Emotional neglect				0.22
40-59 years	0.260 (0.173, 0.347)	0.146 (0.059, 0.233)	0.049 (-0.004, 0.135)	
60-69 years	0.137 (0.034, 0.240)	0.084 (-0.019, 0.187)	0.015 (-0.087, 0.117)	
Sexual abuse				0.04
40-59 years	0.400 (0.274, 0.526)	0.316 (0.190, 0.442)	0.184 (0.060, 0.309)	
60-69 years	0.277 (0.121, 0.434)	0.255 (0.100, 0.411)	0.164 (0.010, 0.319)	
Physical abuse				0.42
40-59 years	0.436 (0.346, 0.526)	0.310 (0.220, 0.400)	0.196 (0.011, 0.285)	
60-69 years	0.382 (0.266, 0.498)	0.319 (0.203, 0.434)	0.216 (0.101, 0.330)	
Emotional abuse				0.004
40-59 years	0.393 (0.297, 0.489)	0.290 (0.194, 0.386)	0.170 (0.074, 0.265)	
60-69 years	0.241 (0.112, 0.369)	0.196 (0.068, 0.324)	0.100 (-0.026, 0.227)	
Cumulative childhood adversity score (0-5)				0.06
40-59 years	0.204 (0.172, 0.236)	0.141 (0.109, 0.173)	0.088 (0.056, 0.120)	
60-69 years	0.145 (0.105, 0.185)	0.109 (0.069, 0.149)	0.072 (0.032, 0.112)	

Abbreviations: CI, confidence interval.
Notes:
Model 1: adjusted for sex.
Model 2: further adjusted for race and ethnicity, educational level, occupation, Townsend deprivation index, maternal smoking around birth, and history of cardiovascular disease and cancer based on Model1.
Model 3: further adjusted for unhealthy lifestyle score based on Model 2.

© 2022 Yang G et al. *JAMA Network Open.*

eTable 4. Associations Between Childhood Adversity and Phenotypic Age Acceleration by Sex

Childhood adversity	Model 1	Model 2	Model 3	P for interaction
	β (95% CI)	β (95% CI)	β (95% CI)	
Physical neglect				0.002
Women	0.382 (0.283, 0.482)	0.225 (0.125, 0.324)	0.206 (0.108, 0.305)	
Men	0.479 (365, 0.594)	0.256 (0.141, 0.370)	0.254 (0.140, 0.368)	
Emotional neglect				0.40
Women	0.200 (0.111, 0.289)	0.110 (0.021, 0.199)	0.023 (-0.065, 0.111)	
Men	0.220 (0.120, 0.320)	0.618 (-0.038, 0.161)	0.007 (-0.091, 0.106)	
Sexual abuse				0.95
Women	0.339 (0.220, 0.459)	0.241 (0.122, 0.360)	0.132 (0.014, 0.250)	
Men	0.324 (0.150, 0.498)	0.230 (0.057, 0.403)	0.146 (-0.026, 0.317)	
Physical abuse				0.003
Women	0.420 (0.321, 0.520)	0.319 (0.219, 0.419)	0.206 (0.107, 0.305)	
Men	0.389 (0.288, 0.489)	0.257 (0.157, 0.357)	0.171 (0.072, 0.270)	
Emotional abuse				0.10
Women	0.300 (0.201, 0.398)	0.200 (0.102, 0.299)	0.096 (-0.001, 0.194)	
Men	0.394 (0.270 ,0.517)	0.220 (0.098, 0.344)	0.140 (0.018, 0.262)	
Cumulative childhood adversity score (0-5)				<0.001
Women	0.164 (0.132, 0.196)	0.110 (0.078, 0.142)	0.066 (0.034, 0.098)	
Men	0.208 (1.066, 1.077)	0.116 (0.076, 0.156)	0.081 (0.041, 0.120)	

Abbreviations: CI, confidence interval.
Notes:
Model 1: no adjustment.
Model 2: adjusted for race and ethnicity, educational level, occupation, Townsend deprivation index, maternal smoking around birth, and history of cardiovascular disease and cancer based on Model 1.
Model 3: further adjusted for unhealthy lifestyle score based on Model 2.

© 2022 Yang G et al. *JAMA Network Open.*

eTable 5. Baseline Characteristics of Participants Excluded Because of Missing Data on Clinical Biomarkers (Used to Calculate Phenotypic Age Acceleration) and the Total Population Who Participated in Both the Baseline Survey and Online Mental Health Survey

Characteristics	Total population N=153,021	Participants excluded N=25,526	P value
Chronological age, years, mean (SD)	56.4 (7.7)	56.2 (7.7)	0.01
Sex, No. (%)			<0.001
Women	86,204 (56.3)	15,225 (59.6)	
Men	66,817 (43.7)	10,301 (40.4)	
Race and ethnicity, No. (%)			0.02
Black	1,065 (0.7)	215 (0.8)	
Chinese	349 (0.2)	61 (0.2)	
Multiple	794 (0.5)	143 (0.6)	
South Asian	1,259 (0.8)	247 (1.0)	
White	148,242 (97.2)	24,627 (96.8)	
Other[a]	837 (0.5)	144 (0.6)	
Educational level[b], No. (%)			0.77
High	70,591 (46.4)	11,697 (46.5)	
Intermediate	50,068 (32.9)	8,207 (32.7)	
Low	31,600 (20.8)	5,231 (20.8)	
Occupation, No. (%)			0.24
Working	97,503 (63.9)	16,308 (64.1)	
Retired	45,077 (29.5)	7,404 (29.1)	
Other	10,122 (6.6)	1,739 (6.8)	
Townsend deprivation index, mean (SD)[c]	-1.7 (2.8)	-1.7 (2.9)	0.01

Abbreviations: SD: Standard Deviation

Notes:

[a]Other includes any races or ethnicities not otherwise specified.

[b]High educational level: college or university degree; Intermediate educational level: A/AS levels or equivalent, O levels/GCSEs or equivalent; Low educational level: none of the aforementioned.

Intermediate educational levels were equivalent to grades 6-12 (O-level equals middle school/junior high, grade 6-8; A-level equals high school, grade 9-12) in the US school system.

[c]For the Townsend Deprivation Index, 0 indicates the mean value for an area, positive numbers indicate lower socioeconomic status, and negative numbers indicate higher socioeconomic status.

© 2022 Yang G et al. *JAMA Network Open.*

eTable 6. Associations of Childhood Adversity (as a Continuous Variable, Range 0-20) With Phenotypic Age Acceleration and Mediation Proportion of Childhood Adversity in Phenotypic Age Acceleration Attributed to Unhealthy Lifestyle

**Childhood adversity**	**Model 1**	**Model 2**	**Model 3**	**Mediation proportion (%) (95% CI)[a]**	**P value**
	**β (95% CI)**	**β (95% CI)**	**β (95% CI)**		
Physical neglect	0.207 (0.170, 0.245)	0.116 (0.079, 0.154)	0.107 (0.070, 0.144)	13.6 (10.4, 18.0)	<0.001
Emotional neglect	0.108 (0.080, 0.137)	0.046 (0.018, 0.075)	0.015 (-0.014, 0.043)	44.0 (35.2, 59.0)	<0.001
Sexual abuse	0.269 (0.215, 0.323)	0.189 (0.136, 0.243)	0.129 (0.076, 0.182)	30.3 (24.5, 38.0)	<0.001
Physical abuse	0.300 (0.259, 0.341)	0.209 (0.168, 0.251)	0.147 (0.106, 0.188)	28.0 (24.2, 33.0)	<0.001
Emotional abuse	0.212 (0.175, 0.249)	0.137 (0.101, 0.174)	0.089 (0.052, 0.125)	31.3 (26.5, 38.0)	<0.001
Cumulative childhood adversity score (0-20)	0.095 (0.083, 0.106)	0.059 (0.047, 0.070)	0.040 (0.028, 0.051)	28.8 (25.5, 33.0)	<0.001

Abbreviations: CI, confidence interval.
Notes:
Model 1: adjusted for sex.
Model 2: further adjusted for race and ethnicity, educational level, occupation, Townsend deprivation index, maternal smoking around birth, and history of cardiovascular disease and cancer based on Model 1.
Model 3: further adjusted for unhealthy lifestyle score based on Model 2.
[a]The model included sex and unhealthy lifestyle score.

© 2022 Yang G et al. *JAMA Network Open.*

eTable 7. Associations of Childhood Adversity With Phenotypic Age Acceleration and Mediation Proportion of Childhood Adversity in Phenotypic Age Acceleration Attributed to Unhealthy Lifestyle in a Complete-Case Sample (N=95,273)

Childhood adversity	Model 1	Model 2	Model 3	Mediation proportion (%) (95% CI)[a]	P value
	β (95% CI)	β (95% CI)	β (95% CI)		
Physical neglect	0.388 (0.301, 0.476)	0.220 (0.132, 0.307)	0.215 (0.128, 0.302)	15.7 (12.0, 20.0)	<0.001
Emotional neglect	0.203 (0.126, 0.279)	0.096 (0.019, 0.173)	0.029 (-0.047, 0.105)	50.3 (38.6, 70.0)	<0.001
Sexual abuse	0.329 (0.216, 0.442)	0.254 (0.141, 0.367)	0.157 (0.045, 0.269)	34.5 (27.8, 45.0)	<0.001
Physical abuse	0.402 (0.321, 0.483)	0.294 (0.214, 0.375)	0.197 (0.117, 0.277)	32.8 (27.4, 40.0)	<0.001
Emotional abuse	0.321 (0.232, 0.410)	0.213 (0.125, 0.302)	0.123 (0.035, 0.211)	31.7 (26.4, 39.0)	<0.001
Cumulative childhood adversity score (0-5)	0.177 (0.149, 0.206)	0.116 (0.087, 0.145)	0.077(0.048, 0.106)	31.0 (26.9, 35.0)	<0.001
0	Ref.	Ref.	Ref.		
1	0.135 (0.060, 0.210)	0.076 (0.001, 0.151)	0.043 (-0.031, 0.117)	35.8 (21.2, 79.0)	<0.001
2	0.273 (0.170, 0.376)	0.158 (0.055, 0.261)	0.076 (-0.025, 0.178)	41.4 (29.5, 66.0)	<0.001
3	0.550 (0.410, 0.690)	0.369 (0.229, 0.509)	0.256 (0.118, 0.395)	28.6 (22.5, 38.0)	<0.001
4	0.701 (0.505, 0.897)	0.464 (0.269, 0.660)	0.310 (0.116, 0.504)	29.7 (23.1, 41.0)	<0.001
5	1.293 (0.920, 1.667)	0.903 (0.530, 1.276)	0.697 (0.328, 1.067)	22.4 (16.5, 32.0)	<0.001

Abbreviations: CI, confidence interval.
Notes:
Model 1: adjusted for sex.
Model 2: further adjusted for race and ethnicity, educational level, occupation, Townsend deprivation index, maternal smoking around birth, and history of cardiovascular disease and cancer based on Model 1.
Model 3: further adjusted for unhealthy lifestyle score based on Model 2.
[a]The model included sex and unhealthy lifestyle score.

© 2022 Yang G et al. *JAMA Network Open.*
